# Resting-state functional connectivity and local activity differences across bothersome and non-bothersome tinnitus phenotypes

**DOI:** 10.3389/fneur.2026.1831863

**Published:** 2026-06-11

**Authors:** Yongpeng Li, Lu Peng, Ying Lan, Binyu Mo, Shihua Yin

**Affiliations:** 1 Department of Otorhinolaryngology-Head and Neck Surgery, Liuzhou People's Hospital Affiliated to Guangxi Medical University, Liuzhou, China; 2 Department of Otorhinolaryngology-Head and Neck Surgery, The Second Affiliated Hospital of Guangxi Medical University, Nanning, China; 3 Department of Otorhinolaryngology-Head and Neck Surgery, The Third Affiliated Hospital of Guangxi Medical University, Nanning, China

**Keywords:** bothersome tinnitus, brain networks, default mode network, functional connectivity, limbic system, resting-state fMRI

## Abstract

**Objective:**

This study aimed to characterize resting-state functional differences across clinically defined bothersome tinnitus, non-bothersome tinnitus, and hospital-based non-tinnitus control groups, focusing on imaging differences related to tinnitus phenotype.

**Methods:**

This case–control study included 61 patients with bothersome tinnitus (BT), 52 with non-bothersome tinnitus (NBT), and 50 hospital-based non-tinnitus controls. Resting-state fMRI scans were acquired, and the data were analyzed using fractional amplitude of low-frequency fluctuations (fALFF), regional homogeneity (ReHo), and seed-based functional connectivity (FC) using broad bilateral temporal lobe regions of interest to assess temporal-related network connectivity, including temporal–limbic interactions. Group comparisons were performed using ANCOVA controlling for demographic, audiological, physiological, sleep-related, and emotional covariates, cluster-level FDR-corrected *p* < 0.05. Exploratory correlation analyses were further conducted to examine associations between extracted imaging indices and clinical measures, as well as potential relationships among significant imaging findings.

**Results:**

Resting-state fMRI revealed significant group differences in functional connectivity between the bilateral temporal lobe seed and the left ACC/mOFC cluster. *Post hoc* analyses showed stronger connectivity in BT than in NBT, whereas BT did not differ significantly from hospital-based non-tinnitus controls. A similar pattern was observed for ReHo in the medial superior frontal gyrus, with lower values in NBT than in both BT and controls. fALFF analyses showed region-specific differences across temporal, frontal, insular, occipital, supramarginal, and postcentral regions, with the most consistent differences observed between BT and NBT. Exploratory analyses showed no significant FC–ReHo correlation within the BT group, and no associations between imaging indices and clinical or audiological measures survived FDR correction. In sensitivity analyses retaining the original covariate structure and additionally adjusting for tinnitus loudness VAS, most BT–NBT differences were attenuated, suggesting that these imaging differences may partly reflect tinnitus loudness or related clinical burden.

**Conclusion:**

Resting-state FC, fALFF, and ReHo measures revealed phenotype-related functional differences between BT and NBT. Because several key measures did not differ between BT and hospital-based non-tinnitus controls, these findings should be interpreted as group-level imaging features across tinnitus phenotypes, not as BT-specific abnormalities.

## Introduction

Tinnitus is the perception of sound in the absence of an external auditory stimulus, a phenomenon often described as ringing or buzzing in the ears (1). It represents a common auditory symptom affecting a substantial portion of the adult population, with reported prevalence rates varying between 4.4 and 26.1% (2, 3). The clinical significance of tinnitus, however, extends far beyond its mere perception. In a subset of patients, the condition evolves into what is clinically defined as bothersome tinnitus (BT), characterized by the persistent and intrusive perception of sound which leads to significant functional impairment (4). Notably, BT affects an estimated 3 to 6% of the general population and is frequently associated with clinically relevant consequences, including emotional distress, cognitive deficits, and sleep disturbances (5–7). This categorization, formally emphasized in the 2014 clinical practice guidelines by the American Academy of Otolaryngology–Head and Neck Surgery, is clinically important because BT necessitates tailored clinical interventions aimed at mitigating distress and improving quality of life, whereas NBT typically exerts minimal impact on daily functioning (4).

Nevertheless, the precise pathophysiological mechanisms underlying tinnitus, particularly the factors associated with tinnitus becoming clinically bothersome, remain inadequately elucidated. Neuroimaging studies have linked tinnitus to abnormal neural activity across distributed auditory and non-auditory brain networks (8, 9), with studies reporting structural and functional alterations in regions including the auditory cortex, limbic system, and prefrontal areas (10–16). Because many studies have analyzed tinnitus as a single condition, it is still unclear which functional changes are related to tinnitus perception and which are related to tinnitus-related distress. Consequently, it remains unclear whether the observed functional reorganizations reflect the mere presence of the phantom sound or are specifically tied to the debilitating emotional and cognitive burden that defines BT.

To address this issue, this study compared resting-state functional measures among clinically defined BT, NBT, and hospital-based non-tinnitus control groups, with a focus on temporal–limbic connectivity and local spontaneous activity. We hypothesized that these measures would differ across tinnitus phenotypes. Given the overlap among tinnitus bothersomeness, loudness, hearing-related burden, and affective symptoms, the analysis was intended to characterize phenotype-related imaging differences rather than to define BT as a categorically distinct neural entity.

## Methods

### Participants

Participants were consecutively recruited between May 2023 and March 2024 from the Inpatient Department of Otolaryngology–Head and Neck Surgery at the Second Affiliated Hospital of Guangxi Medical University. This case–control study included 61 patients with BT, 52 with NBT, and 50 hospital-based non-tinnitus controls (NC). BT and NBT groups consisted of patients with subjective tinnitus. Patients in the BT group exhibited Tinnitus Handicap Inventory (THI) scores **≥** 30, indicating BT, whereas those in the NBT group demonstrated THI scores **<** 30, indicating NBT (17). This cutoff was used to distinguish clinically meaningful tinnitus-related burden from limited functional impact, rather than to define two biologically discrete disease entities. The NC group included individuals without tinnitus symptoms. The NC group was recruited from the same inpatient Department of Otolaryngology–Head and Neck Surgery during the same study period. These participants exhibited no tinnitus symptoms upon clinical screening and were therefore included as hospital-based non-tinnitus controls rather than as community-based healthy volunteers. Anxiety and depression symptoms were assessed using the Self-Rating Anxiety Scale (SAS) and the Self-Rating Depression Scale (SDS), respectively. The exclusion criteria were as follows: (1) History of brain surgery; (2) neurological disorders, such as intracranial tumors, intracranial infections, or cerebrovascular diseases; (3) left-handedness, given its association with differences in brain lateralization; (4) schizophrenia or other severe psychiatric disorders; (5) claustrophobia; (6) objective tinnitus; (7) intermittent tinnitus, defined as episodic tinnitus with symptom-free intervals longer than the periods of tinnitus perception; (8) cranial tinnitus. This study was approved by the Ethics Committee of the Second Affiliated Hospital of Guangxi Medical University (Approval No: 2023-KY(0349)).

### MRI data acquisition

fMRI data were acquired using a 3.0 T MRI scanner (Discovery MR750, GE Healthcare, USA) equipped with a standard 32-channel head coil at the Department of Radiology, Second Affiliated Hospital of Guangxi Medical University. Resting-state functional MRI data were acquired using a gradient echo-planar imaging sequence with the following parameters: repetition time (TR) = 3,000 ms, echo time (TE) = 35 ms, flip angle (FA) = 90°, field of view = 24 cm, matrix size = 64 × 64, slice thickness = 5.0 mm, slice gap = 0 mm, number of slices = 33, bandwidth = 250 Hz, voxel resolution = 3.75 × 3.75 × 5.00 mm^3^, and 128 time points, corresponding to a total acquisition time of 6 min 24 s. During scanning, participants were instructed to remain still, keep their eyes closed, stay awake, and avoid directed thinking to minimize cognitive interference. High-resolution 3D T1-weighted anatomical images were acquired using the T1 BRAVO sequence with the following parameters: TR = 8.1 ms, TE = 3.2 ms, FA = 12°, matrix size = 256 × 224, slice thickness = 1.2 mm, no interslice gap, and voxel resolution = 0.94 × 0.86 × 1.20 mm^3^. These structural images were used for anatomical preprocessing and spatial normalization.

### Preprocessing of fMRI data

Preprocessing of fMRI data was conducted using SPM12 (Statistical Parametric Mapping 12, UK) (18) and RESTplus (version 1.2) (19). The first 10 volumes were discarded to ensure magnetic field stabilization, images underwent slice-timing correction, head-motion realignment, and normalization to MNI standard space using each participant’s T1-weighted structural image, followed by resampling to 3 × 3 × 3 mm^3^ resolution. Participants with excessive head motion (>2 mm translation or >2° rotation) were excluded. Denoising procedures included linear detrending and nuisance regression of Friston-24 head-motion parameters, white matter signal, and cerebrospinal fluid signal. No global signal regression was performed. For FC and ReHo analyses, band-pass filtering (0.01–0.08 Hz) was applied after nuisance regression. ReHo was computed using Kendall’s coefficient of concordance across a 3 × 3 × 3 voxel neighborhood, and the resulting ReHo maps were subsequently smoothed with a 6-mm FWHM Gaussian kernel. For FC, seed-based voxel-wise correlations were calculated after filtering, followed by transformation using Fisher’s r-to-z conversion. fALFF was calculated as the ratio of power within the 0.01–0.08 Hz band to the total power across the detectable frequency range of the BOLD time series. For fALFF and FC analyses, spatial smoothing with a 6-mm FWHM Gaussian kernel was applied after normalization. For ReHo analysis, smoothing was performed only after ReHo map calculation.

### Functional measures and analysis

fALFF, ReHo, and seed-based FC were calculated to characterize local spontaneous activity, local synchronization, and large-scale temporal-limbic connectivity, respectively. Specifically, fALFF was defined as the ratio of power in the low-frequency band (0.01–0.08 Hz) to the total power across the full detectable frequency range determined by the sampling rate, thereby reflecting the relative contribution of spontaneous low-frequency fluctuations to the overall BOLD signal.

ReHo was calculated to assess the local synchronization of blood oxygenation level–dependent (BOLD) signal fluctuations in a 3 × 3 × 3 voxel cluster using Kendall’s coefficient of concordance (KCC). Higher ReHo values indicate greater local neural synchrony. Following map generation, individual ReHo maps were transformed into standardized Z-score maps to promote group-level comparisons. Spatial smoothing (6-mm full-width at half-maximum Gaussian kernel) was applied only after ReHo computation to avoid artificially inflating estimates of local synchrony.

Functional connectivity (FC) was assessed using a seed-based approach with the bilateral temporal lobes as regions of interest (ROIs). These ROIs were anatomically defined using the Automated Anatomical Labeling (AAL) atlas and included temporal regions related to auditory and associative processing, including the superior, middle, and inferior temporal gyri. This seed definition was selected deliberately to capture large-scale auditory-related temporal network interactions with non-auditory regions, particularly limbic and self-referential systems, rather than restricting analysis to narrowly defined primary auditory subregions, including Heschl’s gyrus. For FC analysis, the time series extracted from each seed region was correlated with the time series of all other brain voxels to generate individual FC maps. The resulting correlation coefficients were converted into z-scores using Fisher’s r-to-z transformation to improve normality for subsequent statistical analysis.

### Sample size calculation

*A priori* sample size calculation was conducted using G*Power 3.1.9.7 (*F* tests, ANCOVA: fixed effects, main effects, and interactions) to ensure adequate statistical power. Assuming an effect size of f=0.4 , an alpha level of 0.05, a statistical power of 0.80, a numerator degree of freedom of 2, three groups, and 11 covariates, the minimum required total sample size was estimated to be 64 participants, corresponding to at least 21 participants per group. The final sample size of 163 participants (61 BT, 52 NBT, 50 NC) exceeded this requirement.

### Sensitivity analyses

Sensitivity ANCOVA was performed within the tinnitus cohort to examine whether the BT–NBT differences remained after further accounting for tinnitus loudness. Extracted FC, ReHo, and fALFF values from significant clusters were entered separately as dependent variables, and group was entered as the fixed factor. The original covariate structure of the primary ANCOVA model was retained, including age, sex, years of education, left- and right-ear hearing thresholds, BMI, sleep duration, triglyceride levels, hemoglobin levels, SAS score, and SDS score. Tinnitus loudness VAS score was further added as an additional covariate. The adjusted BT–NBT *p* values were compared with the original *post hoc* results.

### Exploratory combined ROC analysis

Logistic regression–based ROC analyses were performed within the tinnitus cohort to explore whether combined resting-state imaging indices could distinguish BT from NBT. BT/NBT status was entered as the binary dependent variable. Model 1 included the FC index of the left ACC/mOFC cluster and the ReHo index of the bilateral medial superior frontal gyrus. Model 2 further included six selected fALFF indices from significant clusters, including the right middle temporal pole, left medial superior frontal gyrus, right insula, left middle occipital gyrus, right superior frontal gyrus, and right postcentral gyrus. The AUC, 95% CI, optimal cutoff, sensitivity, specificity, and accuracy were calculated. Because the models were developed and evaluated in the same dataset without external validation, the results were interpreted as exploratory apparent performance rather than validated diagnostic accuracy.

#### Statistical analysis

Statistical analyses were performed using RESTplus (version 1.2) and IBM SPSS Statistics (version 25.0) software. Between-group comparisons of fALFF, ReHo, and FC values were performed using analysis of covariance (ANCOVA), controlling for potential confounders, including age, sex, years of education, hearing thresholds, body mass index (BMI), sleep duration, triglyceride levels, hemoglobin levels, and anxiety and depression scores. Covariates were selected according to two principles: (1) inclusion of biologically or methodologically relevant factors that may influence resting-state brain activity in tinnitus, including age, sex, education, left and right hearing thresholds, sleep duration, and affective burden (SAS/SDS); and (2) inclusion of conservative control variables reflecting baseline physiological status in this hospital-based cohort, specifically BMI, triglycerides, and hemoglobin.

*Post hoc* tests were applied to identify specific intergroup differences. Statistical significance was defined as *p* < 0.05, with multiple-comparison correction implemented using a cluster-wise FDR approach. The cluster-defining voxel-level initial threshold was set at *p* < 0.001, uncorrected. Effect sizes for *post hoc* pairwise comparisons were reported using Cohen’s d with 95% confidence intervals.

Exploratory correlation analyses were performed using Pearson’s or Spearman’s correlation tests, as appropriate according to data distribution. These analyses examined associations between selected extracted imaging indices and clinical or audiological measures. The right supramarginal gyrus fALFF index was not included in the exploratory brain–behavior correlation analyses to maintain consistency with the final set of imaging indices retained for subsequent model-based analyses. Because inclusion of this index led to unstable coefficient estimation in the exploratory logistic regression model, it was not retained as a stable candidate imaging feature in the present dataset. Multiple comparisons were controlled using the FDR method.

Bar plots illustrating group differences in fALFF, ReHo, and FC values were generated using R (version 4.3.1) and the ggplot2 (version 3.4.4) package. Brain image visualization was performed using BrainNet Viewer (version 1.7; http://www.nitrc.org/projects/bnv) (20), and the spatial distribution of significant brain regions was mapped in MNI standard space.

## Results

### Demographic and clinical characteristics

The demographic and clinical characteristics of the participants are summarized in Table 1. No statistically significant differences were observed among the three groups (BT, NBT, and NC) in terms of age, gender, years of education, BMI, or hemoglobin levels (*p* > 0.05). However, significant group differences emerged in triglyceride levels, anxiety scores (SAS), depression scores (SDS), and left ear hearing thresholds (*p* < 0.05). The NC group showed measurable hearing thresholds and was therefore not treated as a community healthy control group. This feature was considered in the interpretation of group differences. To reduce the potential influence of these clinical and demographic differences, relevant variables were included as covariates in subsequent functional imaging analyses. Furthermore, as expected, tinnitus severity (assessed by the THI) and tinnitus loudness (VAS) were significantly higher in the BT group compared to the NBT group (*p* < 0.001). Right ear hearing thresholds did not show significant differences among the groups (*p* = 0.103).

**Table 1:** Comparison of clinical characteristics across groups.

Variable	NC (*N* = 50)	NBT (*N* = 52)	BT (*N* = 61)	*p* value
Age (years)	45.8 ± 14.4	44.4 ± 13.5	43.7 ± 16.0	0.752
Sex *n* (%)				0.138
Female	31 (62.0)	22 (42.3)	32 (52.5)	
Male	19 (38.0)	30 (57.7)	29 (47.5)	
Years of education	10.9 ± 4.5	12.2 ± 4.0	11.1 ± 4.3	0.271
BMI (kg/m^2^)	23.3 ± 2.9	24.8 ± 3.9	23.6 ± 3.5	0.080
Hemoglobin (g/L)	135.6 ± 15.1	140.9 ± 18.5	135.7 ± 16.6	0.176
Triglycerides (mmol/L)	1.4 ± 1.3ᵃᵇ	1.7 ± 1.6ᵃ	1.0 ± 0.6ᵇ	0.005*
Sleep Duration (h)	6.0 ± 1.9	6.5 ± 1.7	6.3 ± 1.8	0.448
Anxiety scores	34.0 ± 6.8ᵃ	32.1 ± 5.6ᵃ	38.2 ± 8.9ᵇ	< 0.001*
Depression scores	35.7 ± 7.8ᵃ	35.1 ± 7.5ᵃ	39.2 ± 8.3ᵇ	0.015*
Left ear hearing threshold (dB HL)	26.9 ± 22.6ᵃ	40.9 ± 30.7ᵇ	46.6 ± 32.1ᵇ	0.002*
Right ear hearing threshold (dB HL)	26.8 ± 24.0	32.5 ± 23.9	38.2 ± 33.6	0.103
Tinnitus duration (days)	(−)	29 (6–195)	30 (10–365)	0.138
Laterality of tinnitus, *n* (%)				0.662
Left	(−)	26 (50.0)	32 (52.5)	
Right	(−)	19 (36.5)	18 (29.5)	
Bilateral	(−)	7 (13.5)	11 (18.0)	
Tinnitus loudness VAS score	(−)	3.8 ± 1.5ᵃ	5.9 ± 1.6ᵇ	< 0.001*
Tinnitus handicap inventory scores	(−)	15.9 ± 10.0ᵃ	60.4 ± 16.5ᵇ	< 0.001*

Continuous variables are presented as mean ± standard deviation or median (interquartile range), as appropriate. Groups sharing the same superscript letter (a, b) do not differ significantly at *p* < 0.05. *Tinnitus Duration, Tinnitus Loudness VAS Score, and Tinnitus Handicap Inventory (THI) were only applicable to the BT and NBT groups; therefore, independent two-sample *t*-tests (or non-parametric equivalents) were used to compare these two groups (*p* < 0.05). NC, Hospital-based Non-tinnitus Controls; NBT, Non-bothersome Tinnitus; BT, Bothersome Tinnitus; n (%), number of cases (%); BMI, Body Mass Index; SAS, Self-rating Anxiety Scale; SDS, Self-rating Depression Scale; VAS, Visual Analogue Scale; THI, Tinnitus Handicap Inventory.

### Group differences in temporal-limbic functional connectivity

**Figure 1:**
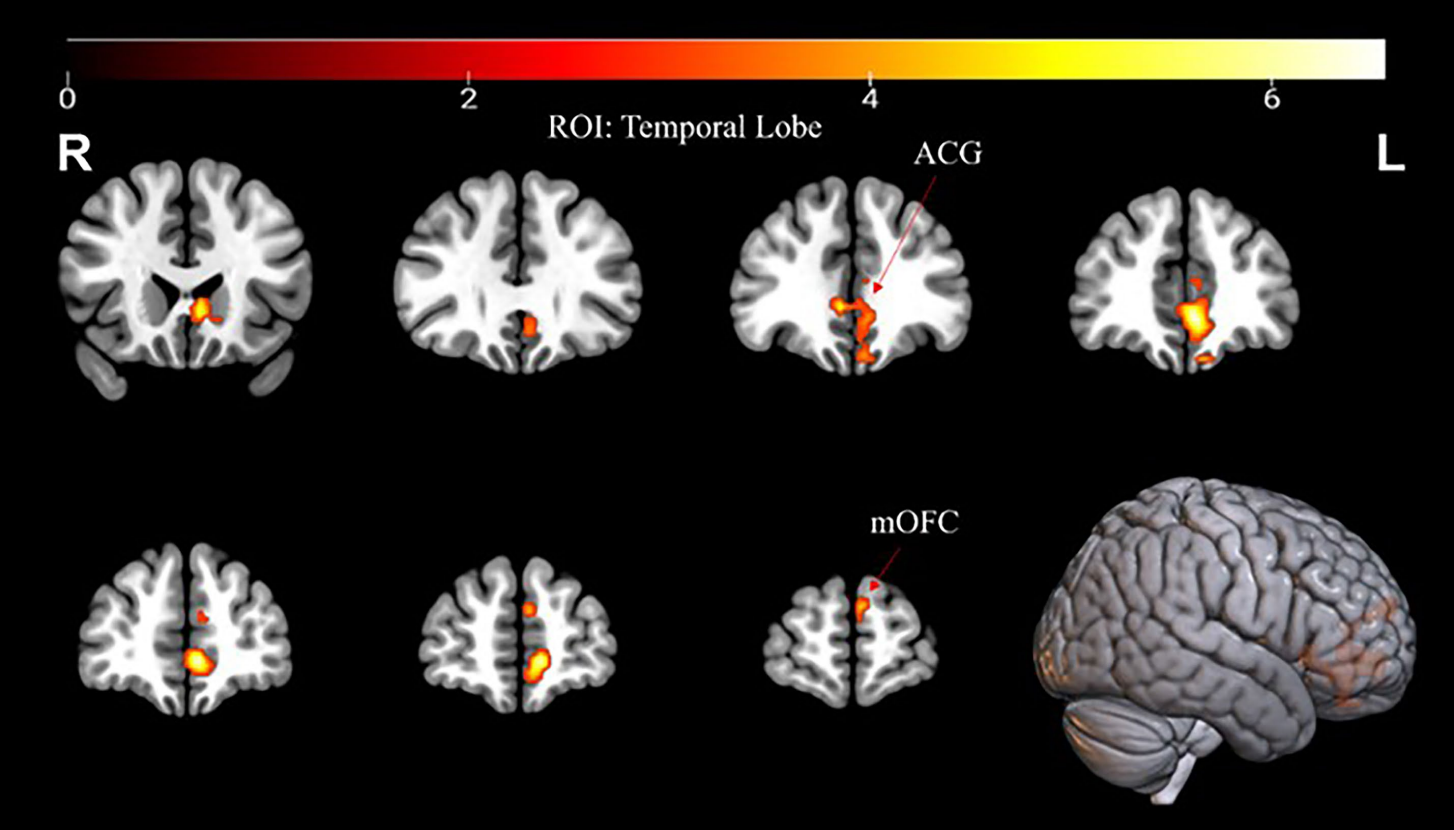
Distribution of brain regions exhibiting clusters with significant FC differences. ACG, Anterior cingulate gyrus; mOFC, Medial orbital frontal cortex; ROI, Region of interest; L, Left; R, Right. The colors on the brain map represent the *F*-value from the ANCOVA. Results were considered significant at the cluster level with *p* < 0.05, FDR-corrected.

**Figure 2:**
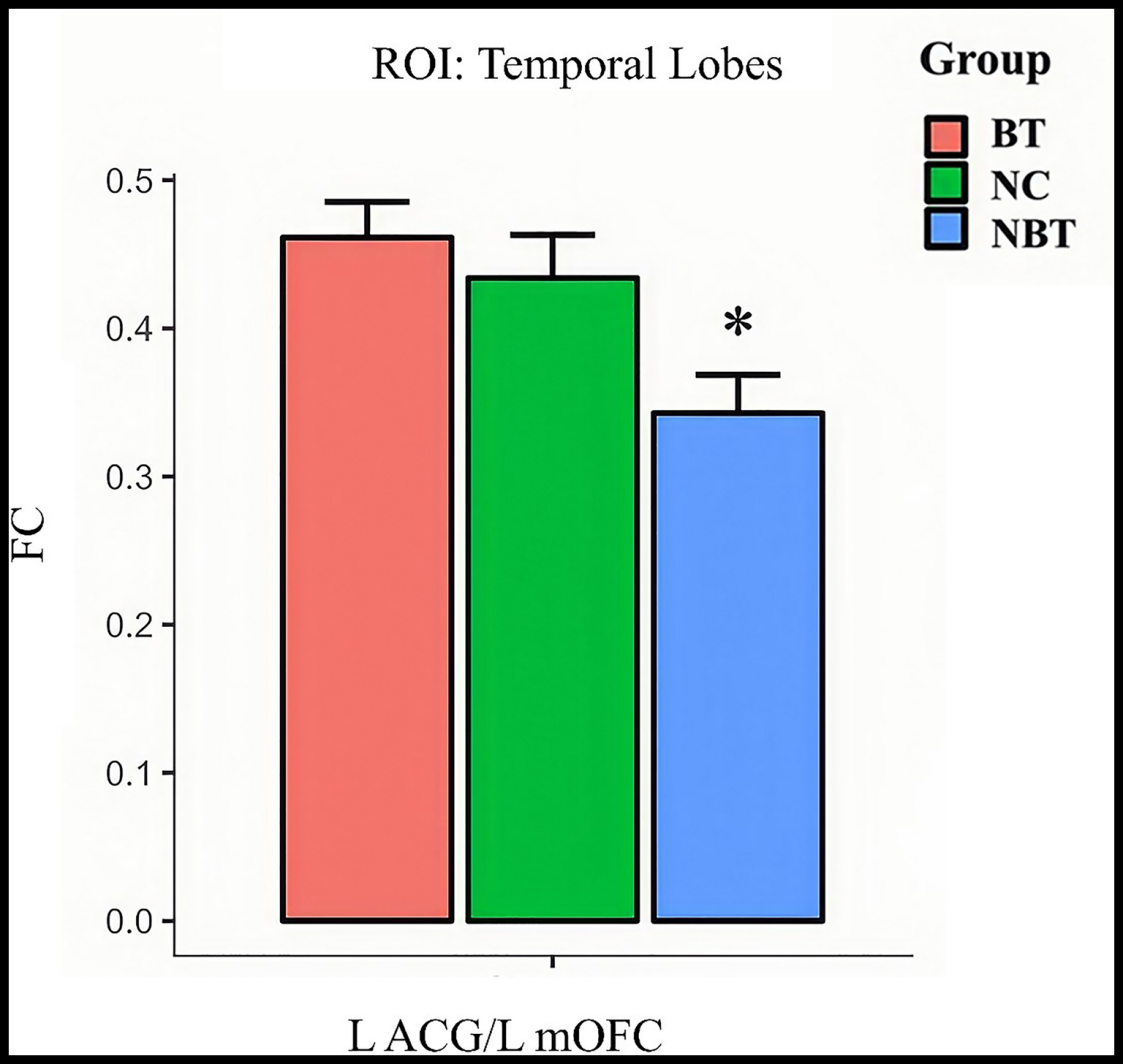
Comparison of FC across BT, NBT, and NC groups. BT, Bothersome tinnitus group; NBT, Non-bothersome tinnitus group; NC, Non-tinnitus control group; FC, Functional connectivity; L ACG, Left anterior cingulate gyrus; L mOFC, Left medial orbital frontal cortex; ROI, Region of interest. Asterisks indicate significant *post hoc* group differences at *p* < 0.05.

Voxel-wise ANCOVA revealed significant group differences in FC between the bilateral temporal lobe seed and a cluster encompassing the left ACC/mOFC. *Post hoc* analyses showed that FC was significantly stronger in the BT group than in the NBT group (*t* = 3.3507, *p* = 0.0033). The NC group also showed stronger FC than the NBT group (*t* = 2.3487, *p* = 0.0312), whereas no significant difference was observed between BT and NC (*t* = 0.7302, *p* = 0.4668) (Tables 2, 3; Figures 1, 2). The FC result was therefore mainly characterized by reduced connectivity in NBT relative to the other two groups.

**Table 2:** Comparison of FC across the BT, NBT, and NC groups.

Brain region	BA	MNI peak coordinates (x, y, z)	*p* value	Cluster size
Left ACC/mOFC	BA 11	−6, 51, −6	0.024	231

BT, bothersome tinnitus; NBT, non-bothersome tinnitus; NC, non-tinnitus control group; ACC, anterior cingulate cortex; mOFC, medial orbitofrontal cortex; FC, functional connectivity; BA, Brodmann area; MNI, Montreal Neurological Institute.

**Table 3:** *Post hoc* tests for functional connectivity.

*Post hoc* tests	*t* value	*p* value	Cluster size	Cohen’s d	95% CI
Left ACG/Left mOFC			231		
BT vs. NC	0.7302	0.4668	231	0.140	−0.235 to 0.514
BT vs. NBT	3.3507	0.0033	231	0.632	0.253 to 1.012
NC vs. NBT	2.3487	0.0312	231	0.466	0.072 to 0.859

LACG, left anterior cingulate gyrus; L mOFC, left medial orbitofrontal cortex; BT, bothersome tinnitus group; NBT, non-bothersome tinnitus group; NC, non-tinnitus control group.

### Region-specific fALFF differences across groups

**Figure 3:**
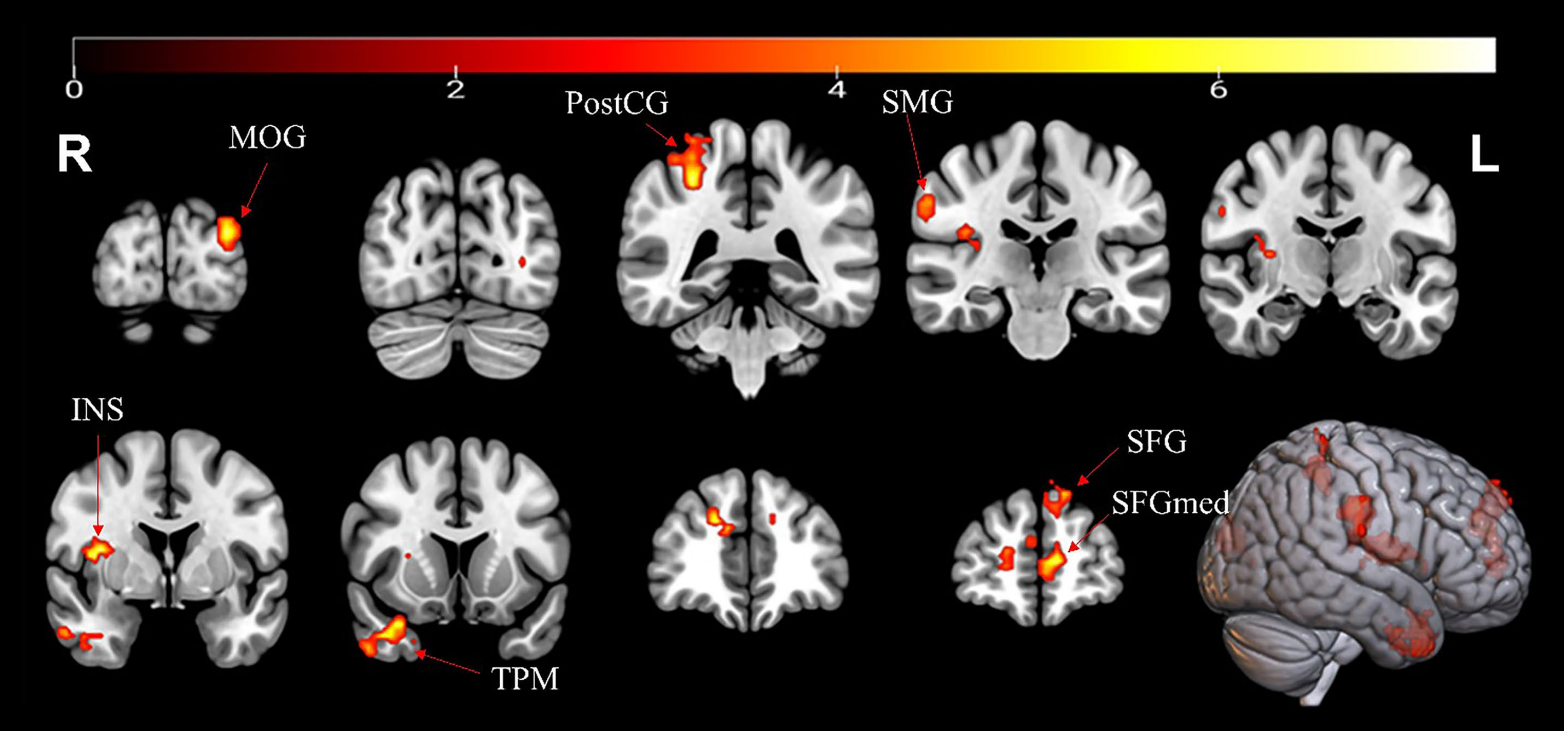
Distribution of brain regions exhibiting clusters with significant fALFF differences. TPM, Temporal pole middle; SFGmed, medial superior frontal gyrus; INS, insula; MOG, middle occipital gyrus; SFG, superior frontal gyrus; SMG, supramarginal gyrus; PostCG, postcentral gyrus; L, left; R, right. The colors on the brain map represent the *F*-value from the ANCOVA. Results were considered significant at the cluster level with *p* < 0.05, FDR-corrected.

**Figure 4:**
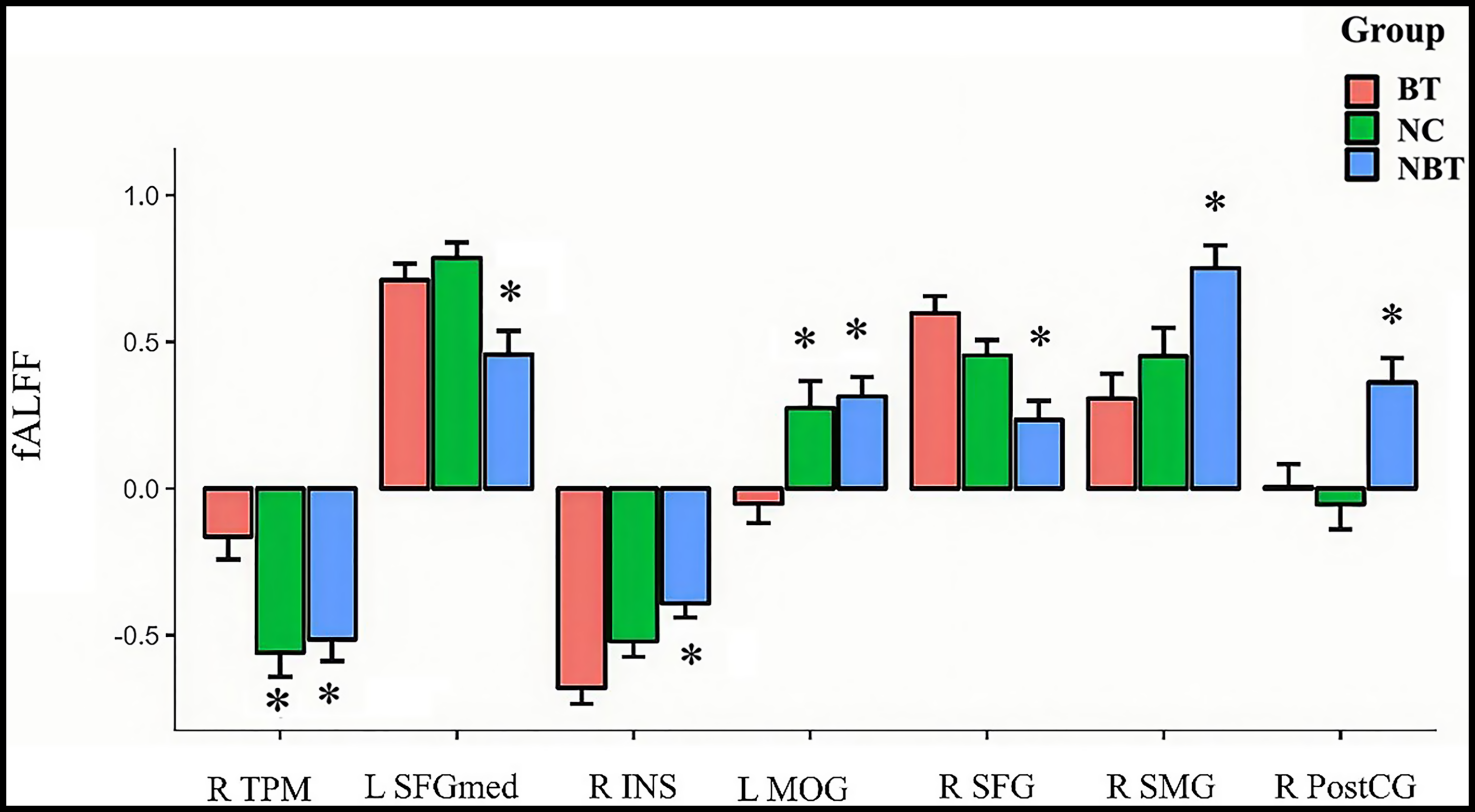
Group-wise comparison of fALFF across BT, NBT, and NC groups. BT, Bothersome tinnitus group; NBT, Non-bothersome tinnitus group; NC, Non-tinnitus control group; R TPM, Right middle temporal pole; L SFGmed, Left medial superior frontal gyrus; R INS, Right insula; L MOG, Left middle occipital gyrus; R SFG, Right superior frontal gyrus; R SMG, Right supramarginal gyrus; R PostCG, Right postcentral gyrus. Asterisks indicate significant post hoc group differences at *p* < 0.05.

Voxel-wise ANCOVA revealed significant group differences in fALFF across seven cortical clusters, including temporal, frontal, insular, occipital, supramarginal, and postcentral regions. *Post hoc* analyses showed that most significant differences occurred between BT and NBT. BT differed from NC only in selected regions, including the right middle temporal pole and left middle occipital gyrus. The right insula showed only a trend-level BT–NC difference and was not interpreted as a significant BT–NC finding (see Figures 3, 4; Tables 4, 5).

**Table 4:** Comparison of fALFF values among the BT, NBT, and NC groups.

Cluster	Brain region	BA	MNI peak coordinates (x, y, z)	*p* value	Cluster size
Cluster 1	Right middle temporal pole	BA38	33, 12, −27	0.002	129
Cluster 2	Left medial superior frontal gyrus	BA10	−12, 48, 27	0.007	141
Cluster 3	Right insula	BA13	39, −21, 21	0.016	105
Cluster 4	Left middle occipital gyrus	BA18/19	−33, −87, 18	0.035	77
Cluster 5	Right superior frontal gyrus	BA10	24, 48, 6	0.026	88
Cluster 6	Right supramarginal gyrus	BA40	63, −18, 36	0.019	85
Cluster 7	Right postcentral gyrus	BA3/2	30, −39, 48	0.033	79

BT, bothersome tinnitus; NBT, non-bothersome tinnitus; NC, non-tinnitus control group; fALFF, fractional amplitude of low-frequency fluctuations; BA, Brodmann area; MNI, Montreal Neurological Institute.

**Table 5:** *Post hoc* tests of fALFF differences.

*Post hoc* tests	t value	*p* value	Cluster size	Cohen’s d	95% CI
Cluster 1: right middle temporal pole			129		
BT vs. NC	3.4696	0.0022	129	0.665	0.278 to 1.046
BT vs. NBT	3.2466	0.0023	129	0.612	0.234 to 0.991
NC vs. NBT	−0.4068	0.6850	129	−0.080	−0.469 to 0.308
Cluster 2: left medial superior frontal gyrus			141		
BT vs. NC	−0.9544	0.3420	141	−0.183	−0.557 to 0.193
BT vs. NBT	2.6433	0.0141	141	0.498	0.123 to 0.875
NC vs. NBT	3.3690	0.0032	141	0.668	0.268 to 1.066
Cluster 3: right insula			105		
BT vs. NC	−2.0656	0.0619	105	−0.395	−0.810 to 0.020
BT vs. NBT	−3.9053	0.0005	105	−0.736	−1.120 to −0.355
NC vs. NBT	−1.8177	0.0721	105	−0.360	−0.751 to 0.031
Cluster 4: left middle occipital gyrus			77		
BT vs. NC	−2.9317	0.0062	77	−0.559	−0.940 to −0.178
BT vs. NBT	−3.8505	0.0006	77	−0.726	−1.109 to −0.345
NC vs. NBT	−0.3495	0.7274	77	−0.069	−0.458 to 0.319
Cluster 5: right superior frontal gyrus			88		
BT vs. NC	1.7993	0.0747	88	0.344	−0.033 to 0.720
BT vs. NBT	4.1453	0.0002	88	0.781	0.398 to 1.166
NC vs. NBT	2.6408	0.0144	88	0.524	0.128 to 0.918
Cluster 6: right supramarginal gyrus			85		
BT vs. NC	−1.1309	0.2606	85	−0.216	−0.591 to 0.159
BT vs. NBT	−3.8095	0.0007	85	−0.719	−1.101 to −0.337
NC vs. NBT	−2.4269	0.0255	85	−0.481	−0.875 to −0.087
Cluster 7: Right postcentral gyrus			79		
BT vs. NC	0.4884	0.6262	79	0.093	−0.281 to 0.467
BT vs. NBT	−3.1062	0.0036	79	−0.586	−0.964 to −0.208
NC vs. NBT	−3.4806	0.0022	79	−0.690	−1.089 to −0.290

BT, Bothersome Tinnitus Group; NBT, Non-bothersome Tinnitus Group; NC, Non-tinnitus Control Group; fALFF, Fractional Amplitude of Low-frequency Fluctuations; R TPM, Right Middle Temporal Pole; L SFGmed, Left Medial Superior Frontal Gyrus; R INS, Right Insula; L MOG, Left Middle Occipital Gyrus; R SFG, Right Superior Frontal Gyrus; R SMG, Right Supramarginal Gyrus; R PostCG, Right Postcentral Gyrus.

### ReHo differences centered on the medial superior frontal gyrus

**Figure 5:**
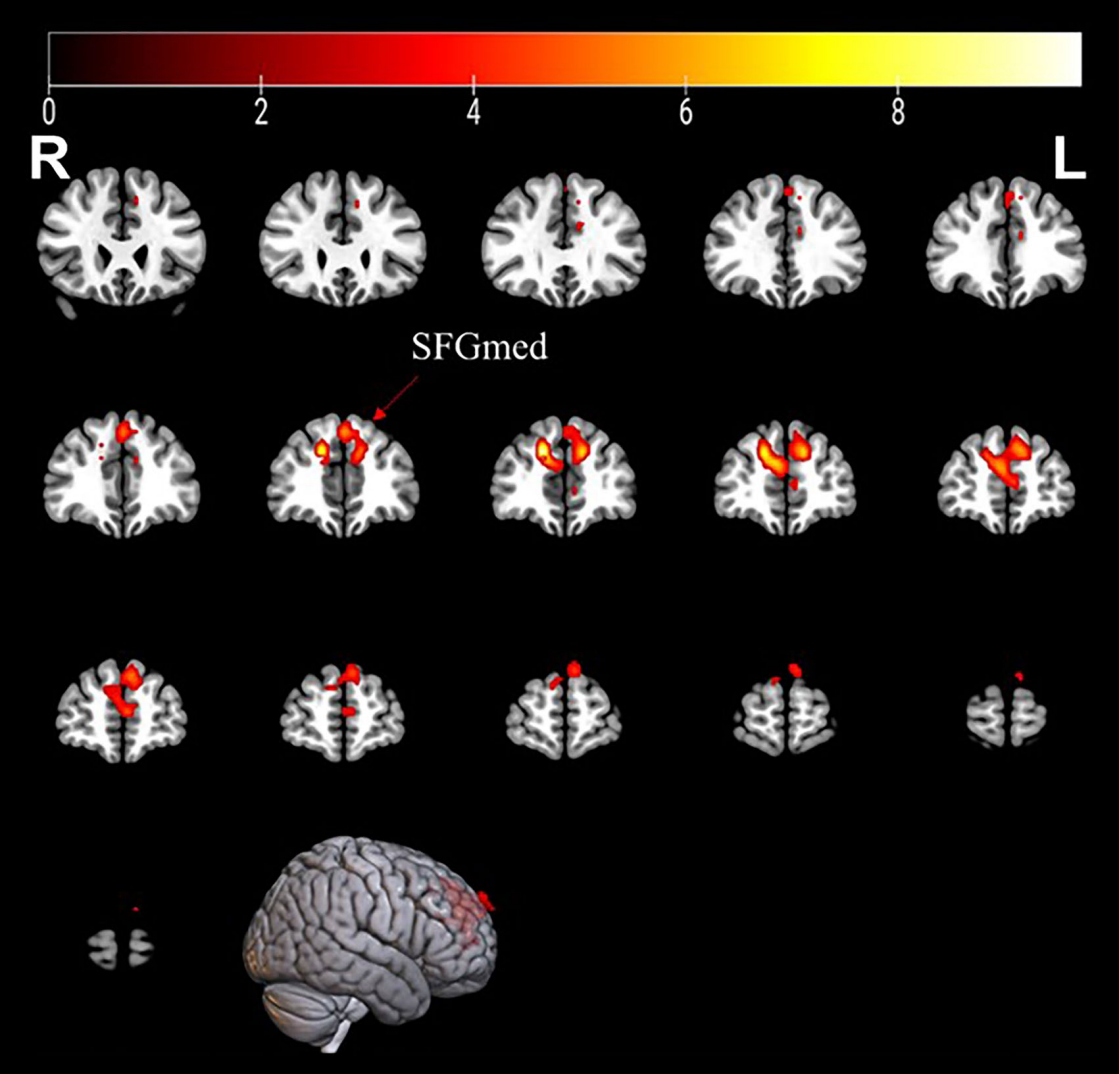
Distribution of brain regions exhibiting clusters with significant ReHo differences. SFGmed, Medial superior frontal gyrus; L, Left; R, Right. The colors on the brain map represent the *F*-value from the ANCOVA. Results were considered significant at the cluster level with *p* < 0.05, FDR-corrected.

**Figure 6:**
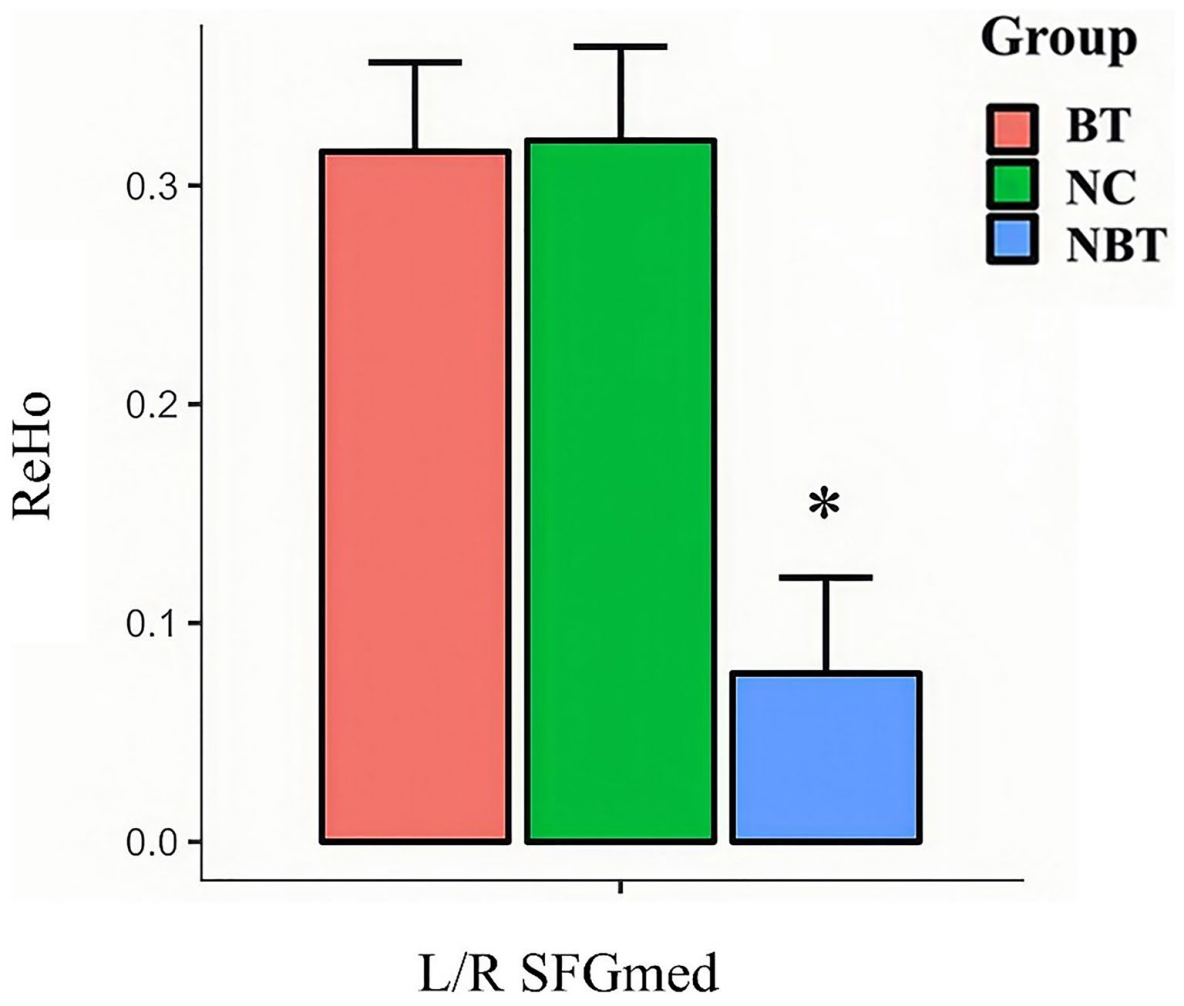
Comparison of ReHo across BT, NBT, and NC groups. BT, bothersome tinnitus group; NBT, non-bothersome tinnitus group; NC, non-tinnitus control group; ReHo, regional homogeneity; L/R SFGmed, left/right medial superior frontal gyrus. Asterisks indicate significant *post hoc* group differences at *p* < 0.05.

Voxel-wise ANCOVA identified significant group differences in ReHo within the bilateral medial superior frontal gyrus. *Post hoc* analyses showed that ReHo was significantly higher in BT than in NBT (*t* = 3.9863, *p* = 0.0002), and also higher in NC than in NBT (*t* = 3.9737, *p* = 0.0002). In contrast, BT and NC did not differ significantly (*t* = −0.0848, *p* = 0.9326) (Tables 6, 7; Figures 5, 6). Thus, the ReHo result was mainly driven by lower values in NBT relative to both BT and NC.

**Table 6:** Comparison of ReHo among the BT, NBT, and NC groups.

Brain region	BA	MNI peak coordinates (x, y, z)	*p* value	Cluster size
Medial superior frontal gyrus	BA10	15, 45, 30	0.003	375

BT, bothersome tinnitus; NBT, non-bothersome tinnitus; NC, non-tinnitus control group; ReHo, regional homogeneity; BA, Brodmann area; MNI, Montreal NEUROLOGICAL Institute.

**Table 7:** *Post hoc* tests for ReHo.

Comparison	*t* value	*p* value	Cluster size	Cohen’s d	95% CI
mSFG			375		
BT vs. NC	−0.0848	0.9326	375	−0.016	−0.390 to 0.358
BT vs. NBT	3.9863	0.0002	375	0.752	0.368 to 1.134
NC vs. NBT	3.9737	0.0002	375	0.787	0.382 to 1.188

BT, Bothersome Tinnitus Group; NBT, Non-bothersome Tinnitus Group; NC, Non-tinnitus Control Group; ReHo, Regional Homogeneity. mSFG, Medial Superior Frontal Gyrus.

### Exploratory brain–behavior correlation analyses

The exploratory brain–behavior correlation results are shown in Supplementary Tables S1–S3. Several nominal associations were observed in the overall tinnitus cohort before FDR correction, mainly involving fALFF measures and FC strength in the left ACC/mOFC cluster. In the BT subgroup, only fALFF in the left middle occipital gyrus was nominally associated with SDS scores. In the NBT subgroup, nominal associations were observed between right-ear hearing threshold and fALFF in the left medial superior frontal gyrus and between left-ear hearing threshold and FC strength in the left ACC/mOFC cluster. However, none of these nominal associations survived FDR correction, suggesting that the identified imaging features were not robustly associated with individual clinical, audiological, or symptom measures.

### Exploratory FC–ReHo correlation analysis

An exploratory analysis was performed within the BT group to examine whether FC strength in the left ACC/mOFC cluster was associated with ReHo values in the bilateral medial superior frontal gyrus. No significant correlation was observed between these measures (Pearson’s *r* = 0.030, *p* = 0.818), suggesting that the FC and ReHo findings were not significantly coupled at the individual level.

### Sensitivity analyses

In the sensitivity analyses performed within the tinnitus cohort, the original covariate structure of the primary ANCOVA model was retained, and tinnitus loudness VAS score was additionally included as a covariate. After this adjustment, most BT–NBT differences were attenuated and were no longer statistically significant. Only the fALFF difference in the left middle occipital gyrus remained significant. These findings suggest that the observed BT–NBT imaging differences may be partly influenced by tinnitus loudness or related clinical burden, and they argue against interpreting these differences as neural correlates of bothersomeness independent of tinnitus loudness. The sensitivity analysis results are presented in Supplementary Table S5.

### Exploratory phenotype-separation ROC analysis

An exploratory logistic regression–based ROC analysis was performed to explore the apparent phenotype-separation performance of combined resting-state imaging indices. Model 1, which included FC strength of the left ACC/mOFC cluster and ReHo values of the bilateral medial superior frontal gyrus, showed limited discrimination, with an AUC of 0.575 (95% CI: 0.466–0.681). At the optimal cutoff of 0.536, the sensitivity, specificity, and accuracy were 0.639, 0.538, and 0.593, respectively.

Model 2 further incorporated six selected fALFF indices, including the right middle temporal pole, left medial superior frontal gyrus, right insula, left middle occipital gyrus, right superior frontal gyrus, and right postcentral gyrus. This model showed moderate apparent discrimination, with an AUC of 0.710 (95% CI: 0.607–0.803). At the optimal cutoff of 0.524, the sensitivity, specificity, and accuracy were 0.738, 0.692, and 0.717, respectively. Compared with Model 1, Model 2 showed an AUC increase of 0.136, with a bootstrap 95% CI of 0.014–0.259 and *p* = 0.027. These findings suggest that region-specific fALFF indices may provide additional information for distinguishing BT from NBT beyond FC and ReHo alone. However, this analysis was exploratory and was not externally validated.

## Discussion

This study identified resting-state functional differences among clinically defined BT, NBT, and hospital-based non-tinnitus control groups. The most consistent differences were observed between BT and NBT, whereas several key FC and ReHo measures did not differ between BT and NC. These findings do not support a simple BT-specific abnormality pattern. Instead, the FC and ReHo results were mainly characterized by lower values in NBT relative to both BT and NC, although this interpretation is limited by the cross-sectional design and the hospital-based nature of the control group.

The pattern in which BT and NC showed comparable FC/ReHo values, whereas NBT showed lower values, should not be interpreted as evidence that BT represents a normalized or healthy neural state. One possible explanation is that NBT may involve lower engagement of temporal–limbic and medial prefrontal systems, consistent with the lower affective salience of tinnitus perception in this group. At the same time, the hospital-based NC group does not provide a normative healthy baseline, which may reduce the apparent contrast between BT and NC. Differences in tinnitus loudness, hearing thresholds, and affective burden between BT and NBT also need to be considered. Therefore, the present findings support a phenotype-level difference but do not establish an adaptive decoupling mechanism.

The inconsistent findings across previous tinnitus fMRI studies may be partly attributable to heterogeneity in tinnitus phenotype, disease duration, hearing status, emotional burden, and analytic strategy (8, 15, 16). Many previous studies treated tinnitus as a single clinical entity, whereas the present study separated BT from NBT according to tinnitus-related functional burden, a distinction emphasized in clinical tinnitus management (4, 7). This distinction may help explain why auditory–limbic and prefrontal–cingulate alterations have not been consistently observed across studies: these effects may vary with tinnitus distress, loudness, hearing impairment, and affective involvement rather than with the mere presence of tinnitus (21–23). In addition, the present cohort consisted largely of recent-onset or subacute tinnitus cases, whereas several previous studies focused on chronic or persistent tinnitus, which may involve different stages of functional reorganization (15, 16). Finally, the broad bilateral temporal lobe seed used in the present study differs from approaches based on more specific auditory, limbic, cingulate, or whole-brain network analyses (14, 21–24). Therefore, the present study does not definitively resolve prior discrepancies, but it suggests that phenotype stratification and careful modeling of hearing and affective factors are important for interpreting tinnitus-related resting-state fMRI findings.

The seed definition should also be considered when interpreting the FC findings. The bilateral temporal lobe ROI included the superior, middle, and inferior temporal gyri and was broader than the primary auditory cortex. Therefore, the present FC result should not be described as highly specific primary auditory cortex–limbic coupling. A more cautious interpretation is that the finding reflects temporal–limbic connectivity, consistent with previous reports implicating auditory–limbic and prefrontal–cingulate–temporal circuits in tinnitus (21–23). Future studies using finer auditory parcellation, such as Heschl’s gyrus, planum temporale, or tonotopically defined auditory cortex, are needed to determine whether the observed effect is specifically auditory or reflects broader temporal associative network involvement.

The distinction between BT and NBT should be interpreted within a broader tinnitus severity continuum, as tinnitus burden often overlaps with loudness perception, hearing-related impairment, sleep disturbance, and affective symptoms (4, 7). In the present sample, BT and NBT differed not only in THI-defined bothersomeness but also in tinnitus loudness, affective burden, and hearing-related variables. Although these factors were statistically adjusted where appropriate, adjustment cannot fully disentangle clinically overlapping constructs. In particular, SAS and SDS may represent components of the BT phenotype rather than merely nuisance variables. Therefore, the adjusted and sensitivity analyses should be regarded as conservative estimates after accounting for tinnitus loudness and general affective burden, rather than evidence that the observed BT–NBT differences represent neural correlates of bothersomeness independent of tinnitus loudness or related clinical burden.

The fALFF findings were region-specific and directionally heterogeneous, involving temporal, frontal, insular, occipital, supramarginal, and postcentral regions. Although this distribution is broadly consistent with previous resting-state studies reporting distributed functional alterations in tinnitus (24–27), the present pattern should not be interpreted as evidence for a unified whole-brain mechanism. The involvement of the temporal pole should also be interpreted in light of its broad connectivity with auditory, paralimbic, visual, and default-semantic networks (28). Overall, these findings suggest region-wise variation in spontaneous activity across tinnitus phenotypes.

Differences in ReHo within the medial superior frontal gyrus may reflect phenotype-level variation in medial prefrontal default-mode systems involved in self-referential and higher-order cognitive processing, although the functional significance of this pattern remains uncertain (29, 30). Similar to the FC findings, ReHo values differed in the BT versus NBT and NC versus NBT comparisons, but not between BT and NC.

The exploratory brain–behavior analyses did not reveal any FDR-corrected association between imaging indices and THI, tinnitus loudness VAS, SAS, or SDS scores. Thus, the FC, fALFF, and ReHo differences identified in this study should be interpreted as group-level imaging features rather than validated symptom-level markers of tinnitus distress. In addition, the exploratory FC–ReHo analysis did not show a significant association within the BT group, suggesting that the temporal lobe–ACC/mOFC connectivity and medial superior frontal ReHo findings may reflect partly distinct group-level imaging features.

These findings may help guide future longitudinal or intervention-based studies by highlighting temporal–limbic connectivity and region-specific fALFF/ReHo differences as measures of interest. However, this framework should not be interpreted as evidence of confirmed maladaptive auditory–limbic coupling, a unified mechanism, or established therapeutic targets. Previous tinnitus neuromodulation studies may provide methodological references for future longitudinal or intervention-based trials (31–33), but the present cross-sectional data cannot determine stimulation parameters or phenotype-specific treatment indications.

An exploratory logistic regression–based ROC analysis was performed to assess the phenotype-separation performance of combined imaging indices. Model 1, including FC and ReHo, showed limited discrimination between BT and NBT, whereas Model 2, which further incorporated selected fALFF indices, showed moderate apparent discrimination. The improved apparent performance of Model 2 suggests that selected fALFF indices may contain additional phenotype-related information, but this result may be influenced by data-driven feature selection and requires validation in an independent cohort.

Several limitations should be noted. First, the NC group was hospital-based rather than community-based and included patients with otological or vestibular disorders. Therefore, it cannot be considered a normative healthy control group. Second, BT and NBT differed in tinnitus loudness and hearing thresholds; although these factors were adjusted for and examined in sensitivity analyses, residual confounding cannot be excluded. Third, the ROC analyses were exploratory and lacked external validation, preventing clinical diagnostic interpretation. Fourth, no FDR-corrected brain–behavior association was identified, limiting symptom-level interpretation. Fifth, the broad bilateral temporal lobe seed reduced auditory subregional specificity.

In conclusion, resting-state FC, fALFF, and ReHo analyses identified functional differences among BT, NBT, and hospital-based non-tinnitus controls. The main differences were observed between BT and NBT, while several key measures did not differ between BT and controls. These findings should be interpreted as group-level imaging differences across tinnitus phenotypes rather than definitive BT-specific neural abnormalities.

## Data Availability

The original contributions presented in the study are included in the article/Supplementary material, further inquiries can be directed to the corresponding author.

## Supplementary material

SUPPLEMENTARY FIGURE S1 ROC curves of exploratory logistic regression models for distinguishing BT from NBT. Model 1 included FC and ReHo indices. Model 2 further incorporated six selected fALFF indices, including the right middle temporal pole, left medial superior frontal gyrus, right insula, left middle occipital gyrus, right superior frontal gyrus, and right postcentral gyrus. Model 2 showed higher apparent discrimination than Model 1.
