# Supplementary figures and images for: Resting-state functional connectivity and local activity differences across bothersome and non-bothersome tinnitus phenotypes
Source: Front Neurol. 2026 Jun 11;17:1831863. doi: 10.3389/fneur.2026.1831863 (PMC13293808; doi:10.3389/fneur.2026.1831863)

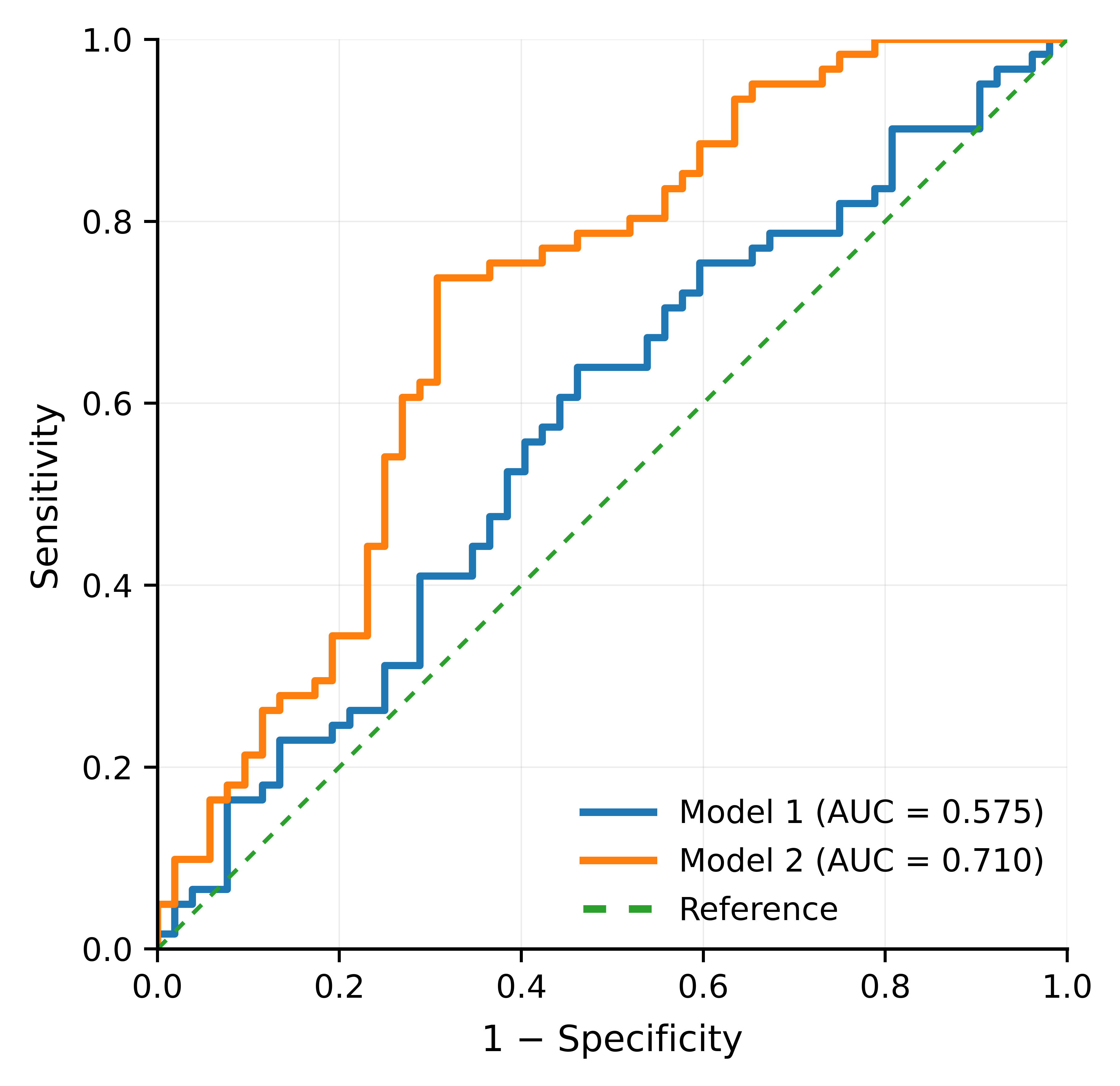

Supplement: SUPPLEMENTARY FIGURE S1 — ROC curves of exploratory logistic regression models for distinguishing BT from NBT. Model 1 included FC and ReHo indices. Model 2 further incorporated six selected fALFF indices, including the right middle temporal pole, left medial superior frontal gyrus, right insula, left middle occipital gyrus, right superior frontal gyrus, and right postcentral gyrus. Model 2 showed higher apparent discrimination than Model 1. [file Image_1.JPEG]
