# Supplementary material for: Resting-state functional connectivity and local activity differences across bothersome and non-bothersome tinnitus phenotypes
Source: Front Neurol. 2026 Jun 11;17:1831863. doi: 10.3389/fneur.2026.1831863 (PMC13293808; doi:10.3389/fneur.2026.1831863)
Supplement: Supplementary file 2 [file Supplementary_file_1.docx]

**Supplementary Material**

**Supplementary Tables S1–S6**

This supplementary file contains aggregate, de-identified supplementary analyses and descriptive summaries prepared for publication with the accepted manuscript. No individual participant identifiers are included.

**Supplementary Table S1. Exploratory brain–behavior correlations in the tinnitus cohort (BT + NBT).**

| **Imaging index** | **Clinical measure** | **r/ρ** | **puncorrected value** | **pFDR-adjusted value** |
| --- | --- | --- | --- | --- |
| fALFF: left medial superior frontal gyrus | Right-ear hearing threshold | -0.26304 | 0.00488 | 0.12591 |
| fALFF: right postcentral gyrus | THI | 0.26094 | 0.00525 | 0.12591 |
| fALFF: left medial superior frontal gyrus | VAS | 0.20917 | 0.02619 | 0.34286 |
| FC: left ACC/mOFC gyrus | SAS | -0.20154 | 0.03231 | 0.34286 |
| fALFF: right postcentral gyrus | VAS | 0.18984 | 0.04401 | 0.34286 |
| fALFF: left middle occipital gyrus | VAS | -0.18698 | 0.04736 | 0.34286 |
| fALFF: right postcentral gyrus | SAS | 0.18484 | 0.05000 | 0.34286 |
| fALFF: left middle occipital gyrus | THI | -0.16204 | 0.08639 | 0.51834 |
| ReHo: bilateral medial superior frontal gyrus | Right-ear hearing threshold | -0.14504 | 0.12534 | 0.61715 |
| fALFF: right postcentral gyrus | SDS | 0.14361 | 0.12915 | 0.61715 |
| fALFF: left medial superior frontal gyrus | THI | 0.13309 | 0.15993 | 0.61715 |
| fALFF: right superior frontal gyrus | Left-ear hearing threshold | 0.12965 | 0.17111 | 0.61715 |
| fALFF: left middle occipital gyrus | SDS | 0.12876 | 0.17408 | 0.61715 |
| fALFF: right insula | Right-ear hearing threshold | 0.12319 | 0.19364 | 0.61715 |
| FC: left ACC/mOFC gyrus | Left-ear hearing threshold | 0.12251 | 0.19610 | 0.61715 |
| fALFF: right superior frontal gyrus | Right-ear hearing threshold | -0.11995 | 0.20572 | 0.61715 |
| fALFF: right insula | THI | 0.11260 | 0.23508 | 0.66375 |
| fALFF: right middle temporal pole | VAS | 0.10699 | 0.25934 | 0.69158 |
| FC: left ACC/mOFC gyrus | THI | -0.09559 | 0.31388 | 0.77023 |
| fALFF: right middle temporal pole | Right-ear hearing threshold | 0.09256 | 0.32952 | 0.77023 |
| fALFF: right insula | VAS | 0.09115 | 0.33697 | 0.77023 |
| fALFF: left middle occipital gyrus | SAS | 0.08019 | 0.39850 | 0.80923 |
| fALFF: left middle occipital gyrus | Right-ear hearing threshold | 0.07498 | 0.42991 | 0.80923 |
| ReHo: bilateral medial superior frontal gyrus | Left-ear hearing threshold | 0.07458 | 0.43239 | 0.80923 |
| fALFF: left medial superior frontal gyrus | Left-ear hearing threshold | 0.07392 | 0.43650 | 0.80923 |
| FC: left ACC/mOFC gyrus | VAS | -0.07363 | 0.43833 | 0.80923 |
| FC: left ACC/mOFC gyrus | Right-ear hearing threshold | -0.06945 | 0.46482 | 0.82635 |
| fALFF: right insula | SAS | 0.06365 | 0.50303 | 0.86234 |
| fALFF: right postcentral gyrus | Left-ear hearing threshold | -0.03010 | 0.75163 | 0.98834 |
| fALFF: right superior frontal gyrus | SAS | -0.02928 | 0.75820 | 0.98834 |
| fALFF: right middle temporal pole | SDS | 0.02899 | 0.76052 | 0.98834 |
| fALFF: right superior frontal gyrus | VAS | 0.02737 | 0.77353 | 0.98834 |
| fALFF: right insula | SDS | -0.02548 | 0.78881 | 0.98834 |
| fALFF: left medial superior frontal gyrus | SAS | 0.02525 | 0.79062 | 0.98834 |
| ReHo: bilateral medial superior frontal gyrus | THI | -0.02391 | 0.80151 | 0.98834 |
| fALFF: right middle temporal pole | Left-ear hearing threshold | -0.02069 | 0.82782 | 0.98834 |
| fALFF: right middle temporal pole | THI | 0.01843 | 0.84636 | 0.98834 |
| fALFF: left medial superior frontal gyrus | SDS | 0.01809 | 0.84916 | 0.98834 |
| ReHo: bilateral medial superior frontal gyrus | SDS | 0.01481 | 0.87631 | 0.98834 |
| ReHo: bilateral medial superior frontal gyrus | SAS | 0.01469 | 0.87731 | 0.98834 |
| fALFF: right postcentral gyrus | Right-ear hearing threshold | 0.01142 | 0.90446 | 0.98834 |
| fALFF: right superior frontal gyrus | SDS | -0.01017 | 0.91486 | 0.98834 |
| ReHo: bilateral medial superior frontal gyrus | VAS | 0.00722 | 0.93950 | 0.98834 |
| FC: left ACC/mOFC gyrus | SDS | -0.00607 | 0.94912 | 0.98834 |
| fALFF: right insula | Left-ear hearing threshold | 0.00591 | 0.95047 | 0.98834 |
| fALFF: right superior frontal gyrus | THI | 0.00234 | 0.98039 | 0.98834 |
| fALFF: left middle occipital gyrus | Left-ear hearing threshold | -0.00146 | 0.98775 | 0.98834 |
| fALFF: right middle temporal pole | SAS | -0.00139 | 0.98834 | 0.98834 |

BT, bothersome tinnitus; NBT, non-bothersome tinnitus; THI, Tinnitus Handicap Inventory; VAS, visual analogue scale; SAS, Self-Rating Anxiety Scale; SDS, Self-Rating Depression Scale; FC, functional connectivity; fALFF, fractional amplitude of low-frequency fluctuations; ReHo, regional homogeneity; FDR, false discovery rate. Correlations were analyzed using Pearson's or Spearman's tests according to data distribution. pFDR-adjusted values were calculated within each supplementary table.

**Supplementary Table S2. Exploratory brain–behavior correlations in the BT subgroup.**

| **Imaging index** | **Clinical measure** | **r/ρ** | **puncorrected value** | **pFDR-adjusted value** |
| --- | --- | --- | --- | --- |
| fALFF: left middle occipital gyrus | SDS | 0.27688 | 0.03076 | 0.75655 |
| fALFF: left middle occipital gyrus | SAS | 0.20642 | 0.11048 | 0.75655 |
| fALFF: right postcentral gyrus | VAS | 0.19988 | 0.12248 | 0.75655 |
| fALFF: left medial superior frontal gyrus | Right-ear hearing threshold | -0.19751 | 0.12707 | 0.75655 |
| FC: left ACC/mOFC gyrus | VAS | -0.19427 | 0.13356 | 0.75655 |
| fALFF: right middle temporal pole | SDS | 0.17809 | 0.16971 | 0.75655 |
| fALFF: right middle temporal pole | Right-ear hearing threshold | 0.17446 | 0.17872 | 0.75655 |
| fALFF: right middle temporal pole | VAS | 0.16646 | 0.19977 | 0.75655 |
| fALFF: right insula | Right-ear hearing threshold | 0.15752 | 0.22536 | 0.75655 |
| fALFF: right postcentral gyrus | SAS | 0.15528 | 0.23209 | 0.75655 |
| FC: left ACC/mOFC gyrus | SAS | -0.15018 | 0.24798 | 0.75655 |
| ReHo: bilateral medial superior frontal gyrus | THI | 0.14696 | 0.25838 | 0.75655 |
| fALFF: left medial superior frontal gyrus | VAS | 0.14150 | 0.27671 | 0.75655 |
| ReHo: bilateral medial superior frontal gyrus | Left-ear hearing threshold | 0.14006 | 0.28166 | 0.75655 |
| fALFF: left middle occipital gyrus | Left-ear hearing threshold | 0.13498 | 0.29965 | 0.75655 |
| fALFF: right superior frontal gyrus | THI | -0.13306 | 0.30666 | 0.75655 |
| fALFF: right postcentral gyrus | Left-ear hearing threshold | -0.13202 | 0.31048 | 0.75655 |
| FC: left ACC/mOFC gyrus | SDS | -0.12768 | 0.32678 | 0.75655 |
| fALFF: right insula | VAS | 0.12217 | 0.34827 | 0.75655 |
| ReHo: bilateral medial superior frontal gyrus | Right-ear hearing threshold | -0.12049 | 0.35497 | 0.75655 |
| fALFF: right superior frontal gyrus | Left-ear hearing threshold | 0.11845 | 0.36326 | 0.75655 |
| ReHo: bilateral medial superior frontal gyrus | VAS | 0.11434 | 0.38025 | 0.75655 |
| fALFF: left middle occipital gyrus | VAS | -0.10935 | 0.40154 | 0.75655 |
| FC: left ACC/mOFC gyrus | THI | -0.10398 | 0.42518 | 0.75655 |
| ReHo: bilateral medial superior frontal gyrus | SAS | 0.10333 | 0.42811 | 0.75655 |
| fALFF: right superior frontal gyrus | Right-ear hearing threshold | -0.10065 | 0.44025 | 0.75655 |
| fALFF: left middle occipital gyrus | Right-ear hearing threshold | 0.10004 | 0.44304 | 0.75655 |
| fALFF: right postcentral gyrus | THI | 0.09894 | 0.44809 | 0.75655 |
| fALFF: right middle temporal pole | THI | 0.09700 | 0.45708 | 0.75655 |
| fALFF: right insula | THI | 0.08210 | 0.52932 | 0.83109 |
| fALFF: right postcentral gyrus | SDS | 0.06892 | 0.59763 | 0.83109 |
| fALFF: right middle temporal pole | Left-ear hearing threshold | 0.06635 | 0.61140 | 0.83109 |
| fALFF: right superior frontal gyrus | VAS | 0.06470 | 0.62032 | 0.83109 |
| fALFF: right insula | Left-ear hearing threshold | 0.06466 | 0.62054 | 0.83109 |
| fALFF: left medial superior frontal gyrus | SAS | 0.06354 | 0.62664 | 0.83109 |
| fALFF: left medial superior frontal gyrus | Left-ear hearing threshold | 0.06093 | 0.64088 | 0.83109 |
| fALFF: right superior frontal gyrus | SDS | 0.05826 | 0.65560 | 0.83109 |
| fALFF: left middle occipital gyrus | THI | 0.05784 | 0.65794 | 0.83109 |
| fALFF: right superior frontal gyrus | SAS | 0.04445 | 0.73376 | 0.89348 |
| fALFF: right insula | SDS | 0.04258 | 0.74457 | 0.89348 |
| fALFF: right middle temporal pole | SAS | -0.03208 | 0.80614 | 0.89562 |
| fALFF: right postcentral gyrus | Right-ear hearing threshold | -0.03030 | 0.81667 | 0.89562 |
| fALFF: left medial superior frontal gyrus | SDS | 0.02961 | 0.82080 | 0.89562 |
| fALFF: right insula | SAS | 0.02544 | 0.84569 | 0.89562 |
| FC: left ACC/mOFC gyrus | Right-ear hearing threshold | 0.02543 | 0.84574 | 0.89562 |
| fALFF: left medial superior frontal gyrus | THI | 0.02197 | 0.86656 | 0.89562 |
| FC: left ACC/mOFC gyrus | Left-ear hearing threshold | 0.02024 | 0.87696 | 0.89562 |
| ReHo: bilateral medial superior frontal gyrus | SDS | -0.00368 | 0.97755 | 0.97755 |

BT, bothersome tinnitus; NBT, non-bothersome tinnitus; THI, Tinnitus Handicap Inventory; VAS, visual analogue scale; SAS, Self-Rating Anxiety Scale; SDS, Self-Rating Depression Scale; FC, functional connectivity; fALFF, fractional amplitude of low-frequency fluctuations; ReHo, regional homogeneity; FDR, false discovery rate. Correlations were analyzed using Pearson's or Spearman's tests according to data distribution. pFDR-adjusted values were calculated within each supplementary table.

**Supplementary Table S3. Exploratory brain–behavior correlations in the NBT subgroup.**

| **Imaging index** | **Clinical measure** | **r/ρ** | **puncorrected value** | **pFDR-adjusted value** |
| --- | --- | --- | --- | --- |
| fALFF: left medial superior frontal gyrus | Right-ear hearing threshold | -0.33256 | 0.01600 | 0.70520 |
| FC: left ACC/mOFC gyrus | Left-ear hearing threshold | 0.30232 | 0.02938 | 0.70520 |
| FC: left ACC/mOFC gyrus | VAS | 0.23773 | 0.08969 | 0.89522 |
| ReHo: bilateral medial superior frontal gyrus | THI | -0.22450 | 0.10959 | 0.89522 |
| fALFF: left medial superior frontal gyrus | SAS | -0.22424 | 0.11002 | 0.89522 |
| FC: left ACC/mOFC gyrus | Right-ear hearing threshold | -0.19416 | 0.16783 | 0.89522 |
| ReHo: bilateral medial superior frontal gyrus | Right-ear hearing threshold | -0.17967 | 0.20247 | 0.89522 |
| fALFF: left medial superior frontal gyrus | VAS | 0.16659 | 0.23785 | 0.89522 |
| fALFF: left middle occipital gyrus | SAS | 0.16649 | 0.23813 | 0.89522 |
| fALFF: right insula | SDS | -0.16388 | 0.24569 | 0.89522 |
| fALFF: right superior frontal gyrus | Left-ear hearing threshold | 0.15511 | 0.27221 | 0.89522 |
| fALFF: right superior frontal gyrus | SAS | -0.15391 | 0.27597 | 0.89522 |
| fALFF: right superior frontal gyrus | Right-ear hearing threshold | -0.14358 | 0.30989 | 0.89522 |
| FC: left ACC/mOFC gyrus | SAS | -0.14006 | 0.32201 | 0.89522 |
| fALFF: right middle temporal pole | SDS | -0.13927 | 0.32477 | 0.89522 |
| fALFF: left middle occipital gyrus | SDS | 0.12804 | 0.36568 | 0.89522 |
| fALFF: right superior frontal gyrus | SDS | -0.12670 | 0.37075 | 0.89522 |
| fALFF: right middle temporal pole | Left-ear hearing threshold | -0.12601 | 0.37338 | 0.89522 |
| fALFF: right superior frontal gyrus | THI | 0.12068 | 0.39408 | 0.89522 |
| fALFF: left middle occipital gyrus | Left-ear hearing threshold | -0.10512 | 0.45830 | 0.89522 |
| fALFF: right insula | Left-ear hearing threshold | -0.09640 | 0.49661 | 0.89522 |
| fALFF: right insula | Right-ear hearing threshold | 0.09496 | 0.50307 | 0.89522 |
| fALFF: left middle occipital gyrus | THI | 0.09033 | 0.52422 | 0.89522 |
| FC: left ACC/mOFC gyrus | THI | 0.08939 | 0.52857 | 0.89522 |
| FC: left ACC/mOFC gyrus | SDS | 0.08608 | 0.54402 | 0.89522 |
| fALFF: right postcentral gyrus | Right-ear hearing threshold | 0.08492 | 0.54944 | 0.89522 |
| ReHo: bilateral medial superior frontal gyrus | SDS | 0.08447 | 0.55160 | 0.89522 |
| fALFF: right insula | VAS | -0.08220 | 0.56236 | 0.89522 |
| fALFF: left medial superior frontal gyrus | SDS | -0.07756 | 0.58471 | 0.89522 |
| ReHo: bilateral medial superior frontal gyrus | SAS | -0.06487 | 0.64777 | 0.89522 |
| fALFF: right postcentral gyrus | Left-ear hearing threshold | 0.05854 | 0.68017 | 0.89522 |
| fALFF: right middle temporal pole | THI | -0.05818 | 0.68203 | 0.89522 |
| fALFF: right middle temporal pole | VAS | 0.05757 | 0.68518 | 0.89522 |
| fALFF: right postcentral gyrus | SDS | 0.05544 | 0.69625 | 0.89522 |
| fALFF: right postcentral gyrus | SAS | 0.05277 | 0.71023 | 0.89522 |
| fALFF: right insula | THI | -0.04923 | 0.72889 | 0.89522 |
| ReHo: bilateral medial superior frontal gyrus | VAS | -0.04769 | 0.73710 | 0.89522 |
| fALFF: left medial superior frontal gyrus | Left-ear hearing threshold | 0.04692 | 0.74118 | 0.89522 |
| fALFF: left middle occipital gyrus | Right-ear hearing threshold | 0.04421 | 0.75562 | 0.89522 |
| fALFF: right superior frontal gyrus | VAS | -0.04156 | 0.76987 | 0.89522 |
| fALFF: right middle temporal pole | SAS | 0.03468 | 0.80718 | 0.89522 |
| fALFF: left middle occipital gyrus | VAS | -0.03285 | 0.81714 | 0.89522 |
| fALFF: right postcentral gyrus | VAS | -0.03277 | 0.81762 | 0.89522 |
| ReHo: bilateral medial superior frontal gyrus | Left-ear hearing threshold | -0.03222 | 0.82062 | 0.89522 |
| fALFF: left medial superior frontal gyrus | THI | -0.02620 | 0.85372 | 0.90271 |
| fALFF: right insula | SAS | 0.02414 | 0.86510 | 0.90271 |
| fALFF: right middle temporal pole | Right-ear hearing threshold | -0.01504 | 0.91574 | 0.93522 |
| fALFF: right postcentral gyrus | THI | 0.00818 | 0.95412 | 0.95412 |

BT, bothersome tinnitus; NBT, non-bothersome tinnitus; THI, Tinnitus Handicap Inventory; VAS, visual analogue scale; SAS, Self-Rating Anxiety Scale; SDS, Self-Rating Depression Scale; FC, functional connectivity; fALFF, fractional amplitude of low-frequency fluctuations; ReHo, regional homogeneity; FDR, false discovery rate. Correlations were analyzed using Pearson's or Spearman's tests according to data distribution. pFDR-adjusted values were calculated within each supplementary table.

**Supplementary Table S4. Admission diagnoses of hospital-based non-tinnitus control participants.**

| **Admission diagnosis** | **n** | **%** |
| --- | --- | --- |
| Sudden sensorineural hearing loss | 27 | 54.0 |
| Vestibular migraine | 6 | 12.0 |
| Benign paroxysmal positional vertigo | 5 | 10.0 |
| Acute vestibular syndrome | 5 | 10.0 |
| Vestibular neuritis | 3 | 6.0 |
| Benign recurrent vertigo | 3 | 6.0 |
| Episodic vestibular syndrome | 1 | 2.0 |

**Supplementary Table S5. Sensitivity ANCOVA for BT vs NBT comparisons.**

| **Imaging index** | **Original p value** | **Sensitivity p value** |
| --- | --- | --- |
| FC: left ACC/mOFC gyrus | 0.0033 | 0.0910 |
| ReHo: bilateral medial superior frontal gyrus | 0.0002 | 0.5852 |
| fALFF: right middle temporal pole | 0.0023 | 0.2667 |
| fALFF: left medial superior frontal gyrus | 0.0141 | 0.3022 |
| fALFF: right insula | 0.0005 | 0.8654 |
| fALFF: left middle occipital gyrus | 0.0006 | 0.0207 |
| fALFF: right superior frontal gyrus | 0.0002 | 0.9303 |
| fALFF: right supramarginal gyrus | 0.0007 | 0.6358 |
| fALFF: right postcentral gyrus | 0.0036 | 0.2791 |

Sensitivity ANCOVA retained the original covariate structure of the primary ANCOVA model and further included tinnitus loudness VAS score as an additional covariate.

**Supplementary Table S6. Exploratory logistic regression–based ROC performance for distinguishing BT from NBT.**

|  | **AUC** | **95% CI** | **Cutoff** | **Sensitivity** | **Specificity** | **Accuracy** | **P value** |
| --- | --- | --- | --- | --- | --- | --- | --- |
| Model 1 | 0.575 | 0.466–0.681 | 0.536 | 0.639 | 0.538 | 0.593 | — |
| Model 2 | 0.710 | 0.607–0.803 | 0.524 | 0.738 | 0.692 | 0.717 | 0.027 |

Model 1 included the FC index of the left ACC/mOFC cluster and the ReHo index of the bilateral medial superior frontal gyrus. Model 2 further included six selected fALFF indices: right middle temporal pole, left medial superior frontal gyrus, right insula, left middle occipital gyrus, right superior frontal gyrus, and right postcentral gyrus. The p value refers to the bootstrap comparison of AUCs between Model 1 and Model 2. AUC 95% CIs and the p value for ΔAUC were estimated using stratified bootstrap resampling with 2,000 iterations.
